# Primary health care reforms in Poland: ambitions versus reality

**DOI:** 10.1093/eurpub/ckac129.626

**Published:** 2022-10-25

**Authors:** A Sagan, K Badora-Musiała, A Domagałaa, I Kowalska-Bobkoa

**Affiliations:** 1 European Observatory on Health Systems and Policies, WHO, London, UK; 2 Jagiellonian University Medical College, Institute of Public Health, Krakow, Poland

## Abstract

Poland, like many other former eastern bloc countries, inherited a health system dominated by narrow medical specialties, a large number of hospital beds and relatively weak primary health care (PHC). Since early 1990s, efforts have been made to strengthen the role of PHC, starting with the introduction of specialization training in family medicine. With privatization of PHC practices the standard of PHC care has improved. However, national audits have repeatedly found PHC to still be inadequate, with the key weaknesses including shortages of family medicine specialists, insufficient provision of preventive services, and limited use of diagnostics, which led to inappropriate referrals and long waiting times for specialist consultations. Between mid-2018 and the end of 2021, a new model of PHC organization was piloted in around 40 PHC practices across Poland that met the model's requirements. The pilot, supported by the World Bank, put much emphasis on health promotion and disease prevention by including health educators and dieticians in PHC teams and by introducing periodic check-ups. It also aimed to reduce referrals to specialist care by allowing PHC doctors to order extensive diagnostic and laboratory tests and, if needed, consult with a range of cooperating specialists. It also sought to increase the role of PHC doctors in the management of chronic conditions by introducing disease management programmes (DMPs) for 11 most prevalent conditions. PHC teams were made responsible for coordinating patients’ care pathways, including post-hospital treatment, and a new role of care coordinator was introduced to that end. After the pilot was concluded, all PHC practices were mandated to hire care coordinators. Implementation of other solutions tested in the pilot remains uncertain, mainly due to the lack of financial and human resources, and the dominance of small PHC practices that struggle to meet the ambitious requirements set out in the new model.

